# Surgical complications of bariatric surgery among patients with rheumatic diseases

**DOI:** 10.22088/cjim.15.1.5

**Published:** 2024

**Authors:** Mohsen Soori, Seyed Hadi Mirhashemi, Fariborz Rashnoo, Gholamhosein Faghih, Fatemeh Ebrahimi, Amir Zamani, Azadeh Hakakzadeh

**Affiliations:** 1 Department of the General Surgery, Loghman Hakim Hospital, Shahid Beheshti University of Medical Sciences, Tehran, Iran; 2 Department of Nutrition, School of Public Health, Iran University of Medical Sciences, Tehran, Iran; 3 Department of Sports and Exercise Medicine, Shahid Beheshti University of Medical Sciences, Tehran, Iran; 4 Physiotherapy Research Center, Shahid Beheshti University of Medical Sciences, Tehran, Iran

**Keywords:** Bariatric surgery, Post-operative complications, Rheumatic diseases, Obesity

## Abstract

**Background:**

Obesity is one the most prevalent diseases all around the world. Some studies have shown a relationship between obesity and the worsening of rheumatic disorders. Higher rates of surgical complications might also be seen among these patients.

**Methods:**

This retrospective-descriptive study was performed on 25 patients with rheumatic disease referred to Loghman Hakim Hospital (Tehran- Iran) and candidates for bariatric surgery (laparoscopic Roux-en-Y gastric and laparoscopic sleeve gastrectomy) from 2018 to 2020. Duration of hospitalization after surgery and history of post-operation surgical and rheumatic complications were assessed. Patients were followed through 6 months after surgery.

**Results:**

The age (Mean±SD) of recruited patients was (38.4 ±10.0) years. The mean body mass index was 45.54 kg/m2 with the minimum and maximum values of 37.5 kg/m2 and 56.5 kg/m2. Among them, the prevalence of rheumatic disorders was rheumatoid arthritis 32%, psoriasis 28%, gout 16%, lupus erythematosus 8%, and other rheumatologic disorders 16%, respectively. One patient had a surgical complication that was a port site infection. One patient had a relapse of gout and other patients had remission and also, their therapeutic drugs were discontinued or reduced.

**Conclusion:**

Patients with rheumatic disorders revealed no higher surgical complication rate after bariatric surgery, and bariatric surgery helped disease remission among these patients.

Obesity is a well-established phenomenon all over the world. According to the World Health Organization, approximately 35% of the world’s population is obese or overweight (1). Obesity is a complex chronic adipose tissue disease and is often associated with other diseases, including rheumatic diseases (2). Obesity may be one of the most critical risk factors for rheumatoid arthritis(RA) (3). A recent meta-analysis showed that with the onset of rheumatoid arthritis among obese people, the chances of recovery are 43 percent, and the chances of long-term remission are 51 percent (4). 

Recently, bariatric surgery has been known as the most successful and reliable weight-loss treatment method (5). The significant weight loss after bariatric surgery may improve musculoskeletal disorders outcomes (6). Previous studies have shown different outcomes of bariatric surgery among patients with different rheumatic diseases. Assessing the relationship between obesity and RA, unresolved questions and paradoxes (7, 8). Patients with psoriatic arthritis are more likely to be obese than patients with psoriasis. In fact, obesity is not only a comorbid disease in psoriatic arthritis but also a real risk factor that has been shown in various studies (9, 10). 

In addition, weight loss of 5% or more from baseline body weight was found to slow the progression of the disease over the next six months, and weight loss of more than 10% was associated with a sharp decline in the disease progression from the beginning of psoriasis arthritis (11). Assessing the effects of bariatric surgery on lupus found that about three years after the surgery, about half of patients could reduce their biologic drugs (12).

Bariatric surgeries are usually safe and successful, but severe complications may be associated. Leaks, stenoses, bleeding, and venous thromboembolic events are early complications (13). About complications of bariatric surgery in rheumatic patients, it has also been observed that acute gout attacks occur after bariatric surgery. A faster and greater decrease in serum uric acid levels in the early post-operative period may trigger gout attacks (14, 15) however, there are not many studies on different types of rheumatic and non-rheumatic complications after bariatric surgery among these patients. Therefore, considering this and the rising incidence of bariatric surgeries as a definitive treatment of obesity, we aimed to investigate the frequency of surgical complications after bariatric surgery among patients with rheumatic diseases.

## Methods

A retrospective descriptive study was performed in Loghman Hakim Center in Tehran (Iran). The ethical committee approved this study of Shahid Beheshti University of Medical Sciences (IR.SBMU.MSP.REC.1399.222). After ethical approval for the study was obtained, the patients were given a clear explanation of the study’s objectives, and all patients provided written consent. Among all patients referred to this center and were candidates for bariatric surgery (laparoscopic Roux-en-Y gastric and laparoscopic sleeve gastrectomy) due to obesity from 2018 to 2020, patients with any rheumatic disorders were extracted. The basis for each patient’s disease diagnosis was preoperative tests and the patient’s history. Of 1400 patients who underwent bariatric surgery, twenty-five patients had obesity concurrent with rheumatic diseases. They were contacted, and after receiving consent forms from patients, we asked them about age, date of bariatric surgery, duration of hospitalization, drug history, and history of any post-operative or rheumatic-related complications. They were also requested to come periodically for follow-up visits in the hospital through 1, 3, and 6 months after surgery. Surgical complications such as wound infection and mass palpation in the abdomen were evaluated. We asked them about their rheumatic disease symptoms change individually after the sixth month of collecting these data from recruited patients. Mean and standard deviation of numerical demographic data are reported and for categorical data, frequency and percent are reported. The collected descriptive data were analyzed using SPSS version 20.

## Results

Of the 1400 patients reviewed, 25 (1.7%) had a diagnosis of rheumatic diseases. The mean age of recruited patients was 38.4 years. The youngest and oldest were 20 and 58 years old. The mean body mass index was 45.54 kg/m2 with the minimum and maximum values of 37.5 kg/m2 and 56.5 kg/m2, respectively. The average number of hospitalization days per person was 4.04 days, which varied from 3 to 6 days (table 1). Regarding about frequency of rheumatic diseases among these patients, rheumatoid arthritis, psoriasis arthritis, gout, and lupus erythematosus had the highest prevalence of 32%, 28%, 16%, and 8%, respectively. Other rheumatic diseases were seen in 16% of patients with osteoarthritis, fibromyalgia, and RA associated with psoriasis (table 2). 

All patients with different kinds of rheumatic diseases were treated medically. Non-steroidal anti-inflammatory drugs (NSAIDs) were the most prevalent drugs used. Prescribed medication among the patients before surgery is shown in table 3.

In terms of bariatric surgery method, laparoscopic Sleeve Gastrectomy was performed for twenty-four patients, and only one patient was operated on with laparoscopic Roux-en-Y gastric bypass. One patient had a port site infection on the third day after surgery. He only took NSAIDs and did not take prednisolone or any other drugs before surgery. The cellulitis around the port site remained after antibiotic therapy with cefazolin sodium (Keflin) which was started after surgery. Then cefazolin sodium was changed to vancomycin 1 gram twice in day. And after three days, he was discharged with a good condition without any other problems. Also in another patient who had lupus erythematosus, leak symptoms were notices one day after surgery. He was admitted to the surgical important care unit ward (ICU), it was demonstrated there was no leak and it was atelectasis. He was also discharged with good condition on sixth days after surgery.

In this study, one patient had a relapse. A 34-year-old man with gout showed disease relapse one month after surgery. He experienced a gout attack, but other patients did not relapse after bariatric surgery. Furthermore, their rheumatic-related medications were reduced in dose at the end of the study. After six months for all patients, prednisolone, NSAIDs, methotrexate, and Sinora were continued for all, and other drugs such as sulfasalazine and hydroxychloroquine were discontinued by their rheumatologists (table 3). All of the patients were satisfied with the result of bariatric surgery. 

**Table 1:** Descriptive charasterictics of patients

**Variable**	Mean ± SD*	**(Min - Max)**
**Age(year)**	38.4 ± 10.02	(20 - 58)
**BMI(kg/m** ^2^ **)**	45.54 ± 4.76	(37.5 - 56.5)
**Weight (kg)**	125.15 ± 21.18	(96 - 173)
**Height (cm)**	165.33 ± 8.1	(153 - 180)
**Hospitalize (day)**	4.04 ± 0.98	(3 - 6)

*SD: standard deviation, BMI: Body mass index

**Table 2:** Frequency (%) of rheumatic diseases in patients before bariatric surgery

**Rheumatic disease**	**Frequency (%)**
RA*	8 (32%)
**psoriasis**	7 (28%)
**gout**	4 (16%)
**lupus erythematosus**	2 (8%)
**others**	4(16%)

*Rheumatoid arthritis

**Table 3:** Frequency distribution of prescribed medications in patients with rheumatic diseases before bariatric surgery

**Medication**	**Frequency (%)** **Before Surgery**	**Frequency (%)** **After Surgery**
NSAIDs*	10 (40%)	2 (8%)
**Prednisolone**	8 (32%)	3 (12%)
**Methotrexate**	7 (28%)	2 (8%)
**Hydroxychloroquine**	7 (28%)	0
**Allopurinol**	3 (12%)	2 (8%)
**Colchicine**	2 (8%)	2 (8%)
**Sulfasalazine**	2 (8%)	0
**Sinora**	2 (8%)	0

*Nonsteroidal anti-inflammatory drugs

## Discussion

This study evaluated 25 patients with rheumatic diseases who underwent bariatric surgery. In 96% of them, after six months from bariatric surgery, the rheumatologist reduced their rheumatic drugs. In Sparks et al study it was mentioned that rheumatoid arthritis was remitted after 12 months from bariatric surgery (16). The activity of RA disease was improved dramatically during post-surgical visits among patients in our study. Also use of RA-related drugs was reduced. Although our study was done during only 6 months for follow-ups and with fewer numbers of participants but results are similar (16).

In Farias et al study, ten patients with psoriasis were evaluated after bariatric surgery (17). The mean BMI was 38.8 ± 5.2 kg/m2. For 80% of the patients, Roux-en-Y gastric bypass was performed. About 80% of patients discontinued their medication, and 70% experienced remission (17). In the current study, only one patient showed exacerbated symptoms and had gout. In patients with psoriasis, none of them experienced relapsing. The types of bariatric surgeries in these two studies were different. In our study, sleeve gastrectomy was performed for 95% of patients, but in Farias et al study, it was done on 20% of patients. Our patients had higher BMI (45.54 ± 4.76 Kg/m2), but in Farias et al study, it was 38.8 ± 5.2 kg/m2. Comparing these two studies showed that the different types of bariatric surgery in patients with the rheumatic disease have similar results, approximately (18).

Romero-Talamás et al conducted a study on psoriatic patients who underwent bariatric surgery (18). They found that about 25% of patients did not take any medications after bariatric surgery at the end of follow-up visits. Forty percent had remission in their disease, and only one patient experienced relapse post-operation. In the present study, all of the patients continued all drugs except NSAIDs and prednisolone for them with a lower dose than before. In our study, one patient had a relapse, but he did not have psoriasis. All patients with psoriasis had remission. Our study was performed for six months, but Romero-Talamás et al study’s mean follow-up time was 26.2±20.3 months, and maybe if the duration of our study were longer, our patients would experience a complete hold in their treatment drugs (19).

Xu et al performed a study on obese patients with RA (19). They found a significant reduction in the use of leflunomide, biologic drugs, combination therapies, and NSAIDs after 12 months. Also, they found that weight loss following bariatric surgery was associated with less disease activity in RA patients with obesity. These findings were similar to our study results (19).

Corcelles et al conducted a study on patients with lupus erythematosus after bariatric surgery (20). They observed that 13% of the patients had post-operative complications, and reoperation was performed. They found that post-operative complications are related to immunosuppressive therapy. About 77% of the patients were taking immunosuppressive medications in their study. In the current study, about 65% of recruited patients took immunosuppressive medications, but we did not observe any post-operation complications. One patient with lupus erythematosus had atelectasis after surgery that was unrelated to post-bariatric surgery complications. Maybe a lower rate of post-operation complications in our study is due to the type of surgery. In Corcelles et al study, the most prevalent type of surgery was Roux-en-Y gastric bypass. However, in the present study, it was sleeve gastrectomy. In that study they found, about 40% of patients experienced remission in their disease but in the current study we observed only one patient had a relapse in disease. This difference could be explained with the follow-up time. In the present study, we had a lower duration of follow-up visits in comparison to the Corcelles et al study (21). 

In the average population, surgical complications in bariatric surgery occur in about 4.5% of cases. In our study, we saw that only one patient had a surgical complication (4%). It demonstrates no difference between complication rates in the average population and patients with rheumatic diseases (21).

Bariatric surgery in patients with rheumatic diseases can be a type of treatment. Also, rheumatologists can reduce drugs and even hold them after bariatric surgery, especially laparoscopic sleeve gastrectomy. Bariatric surgery in patients with rheumatic diseases has a complication rate similar to the average population. According to this study and others, patients affected by gout disease need more accurate and close follow-ups after surgery. Further studies investigating outcomes of obese patients with gout disease before and after bariatric surgery are yet needed.
